# The argument against the use of dupilumab in patients with limited polyp burden in chronic rhinosinusitis with nasal polyposis (CRSwNP)

**DOI:** 10.1186/s40463-023-00668-z

**Published:** 2023-09-28

**Authors:** Scott A. Hardison, Brent A. Senior

**Affiliations:** 1 Department of Otolaryngology/Head and Neck Surgery, The University of North Carolina at Chapel Hill, Chapel Hill, NC USA; 2 Division of Rhinology, Allergy and Endoscopic Skull Base Surgery, The University of North Carolina at Chapel Hill, 101 Manning Drive, Chapel Hill, NC 27599-7070 USA

**Keywords:** Chronic rhinosinusitis, Endoscopic sinus surgery, Eosinophilic rhinitis, Nasal polyposis, Quality of life, Sinusitis

## Abstract

Dupilumab and other biologics have revolutionized the management of recalcitrant polyps in patients with chronic rhinosinusitis with nasal polyposis (CRSwNP). Despite strong evidence for the efficacy of dupilumab in treating polyps, factors such as cost and uncertain efficacy over surgery have limited its use to patients who have failed the use of topical nasal steroids and initial surgical management. Likewise, the use of this drug is often directed towards patients with greater polyp burdens. Recent studies, however, have investigated the use of dupilumab and other biologics in expanded patient populations, including those with limited polyp burden. The overall trend in the literature suggests a future move towards the use of biologics as first-line therapy for all patients with CRSwNP. The arguments against widespread, routine use of dupilumab and biologics in all patients with CRSwNP are threefold. First, endoscopic sinus surgery has been found to provide similar symptomatic benefit to dupilumab in the treatment of these patient populations. The surgical improvement of patients’ sinonasal anatomy offers a rapid elimination of sources of ongoing inflammation that contribute to long-term polyp formation and symptoms. Medical non-compliance in this specific patient population is known to be an issue, with surgery offering a much greater long-term prospect of symptomatic relief in non-compliant patients. The second concern revolves around the potential for side effects of dupilumab and other biologics. Initial studies have shown an acceptable safety profile, but trials assessing the use of dupilumab for a separate indication revealed a higher rate of conjunctivitis. Long-term safety data is limited for biologics, and we must be prepared for the possibility of severe, unanticipated adverse events in the future. Our third and most profound concern is the significant cost of dupilumab. This medication is enormously expensive, and all current literature suggests that treatment would need to be life-long to remain effective. Studies comparing endoscopic sinus surgery to various biologics, including dupilumab, have shown comparable overall quality of life metrics with biologics, all while delivering considerably higher anticipated lifetime costs. As our knowledge progresses regarding the efficacy of dupilumab and other biologics in a variety of clinic situations, it is important to understand the context in which these advances are being made. While dupilumab and other biologics offer undeniable efficacy in the treatment of chronic rhinosinusitis with nasal polyposis which has failed to respond to standard therapies, we argue that biologics remain only a component of effective management in this patient population. Endoscopic sinus surgery and topical nasal steroids offer equal efficacy and substantially lower costs than biologics, and these factors should be considered when selecting treatment options for patients.

## Background

Chronic rhinosinusitis with nasal polyposis (CRSwNP) is a classification of chronic rhinosinusitis (CRS) characterized by the presence of polypoid changes to the nasal mucosa. The underlying etiology behind polyp formation can vary widely, including aspirin-exacerbated respiratory disease (AERD), allergic fungal sinusitis (AFS) and cystic fibrosis (CF), with many cases ultimately idiopathic [1]. Though much remains unknown about the exact biochemical pathways for polyp formation, it is clear that T-helper 2 (Th2) inflammation and its associated cytokines, interleukin-4 (IL-4), IL-5 and IL-13, play a significant role [1]. Initially developed for use in treating asthma and eczema, monoclonal antibodies targeting IgE (omalizumab) and Th2 cytokines (dupilumab and mepolizumab) have also proven effective at combatting nasal polyps [2–5]. These drugs, known broadly as biologics, have greatly expanded treatment options for patients with recalcitrant nasal polyps. In particular, dupilumab (anti IL-4 receptor α) has been the focus of a number of landmark clinical trials and gained approval by various regulatory bodies for use in patients with CRSwNP [3–5].

To this point, dupilumab has been primarily utilized in the treatment of patients with CRSwNP who have failed oral steroids, topical steroids and surgical management [4]. Recent studies have sought to broaden the population of patients who could potentially receive dupilumab and other biologics. One such study, authored by our colleagues at Medical University of Vienna (Campion et al.), appears in this issue of the Journal of Otolaryngology Head and Neck Surgery. This interesting study found that polyp size at the initiation of dupilumab therapy had no impact on the efficacy of treatment, as measured by rate of change in Total Polyp Score (TPS), smell identification and the Sino Nasal Outcome Test (SNOT-22) quality of life questionnaire. They also found that the concomitant use of oral and topical steroids during dupilumab therapy had no effect on treatment outcomes.

We feel that it is important to consider this study in the context of current regulations, comparable efficacy of surgery, cost, adverse effects and current practice guidelines. Below, we present our argument against the routine use of dupilumab in patients with limited polyp burden in CRSwNP.

### Current regulations

On June 16, 2019, the United States Food and Drug Administration (FDA) approved dupilumab for use in patients 18 years and older with CRSwNP [6]. This approval took the form of an amendment to the list of indications for this drug. The FDA’s indication for CRSwNP specifies that the drug may be used “as an add-on maintenance treatment in adult patients with inadequately controlled chronic rhinosinusitis with nasal polyposis (CRSwNP) [7]”. Following the approval of dupilumab, two other biologics have been approved by the FDA for recalcitrant CRSwNP; omalizumab and mepolizumab.

In the European Union (EU), pharmaceuticals are regulated by the European Commission (EC). On October 29, 2019, the EC approved dupilumab for use in adults with *severe* CRSwNP. The European Commission’s indication closely mirrors that published by the US FDA, stating that dupilumab is indicated “as an add-on therapy with intranasal corticosteroids for the treatment of adults with severe CRSwNP for whom therapy with systemic corticosteroids and/or surgery do not provide adequate disease control [8]”. This language is more explicit than that offered by the US FDA, essentially outlining the expected treatment algorithm for patients with CRSwNP and making it clear that dupilumab is to be offered to truly recalcitrant patients.

In summary, review of regulations in both the Unites States and European Union indicates that dupilumab is indicated for recalcitrant disease and is considered an “add-on” therapy. Therefore, use of dupilumab as first-line therapy without concomitant use of intranasal steroids would be considered off-label use. The inherent legal, practical and ethical considerations of off-label use of dupilumab must be weighed against aggressive use of this drug.

### Comparable efficacy of surgery

While the efficacy of dupilumab and other biologics has been well-established for patients with CRSwNP who have persistent symptoms after failure of initial surgical management and steroids [3, 4], the central question is whether biologics offer equal treatment efficacy to endoscopic sinus surgery (ESS). This is an especially important topic to understand, as non-surgical healthcare professionals may inherently seek to offer biologics in place of surgical management. All physicians (including otolaryngologists) tend to offer therapeutic options based on both their training background and the treatments that they themselves are able to provide [9].

The topic of the efficacy of ESS versus biologics was recently studied in a multi-center prospective cohort, conducted by Miglani et al. at the Medical University of South Carolina and Oregon Health Sciences University [10]. A total of 111 patients were divided into cohorts of patients receiving ESS, dupilumab, mepolizumab or omalizumab [10]. A variety of objective and subjective outcome measures were assessed at 24 and 52 weeks [10].

At 24 weeks, patients undergoing ESS displayed significantly greater improvements in the Sino Nasal Outcome Test (SNOT-22) and significantly lower nasal polyp scores (NPS) compared to dupilumab (*p* < 0.05, *p* < 0.001 respectively) and omalizumab (*p* < 0.001 and *p* < 0.001 respectively) [10]. The study found comparable improvements in smell identification between those receiving ESS and all biologics (*p* > 0.05) [10].

At 52 weeks, improvement in SNOT-22 scores were comparable between ESS and dupilumab (*p* = 0.21), but NPSs were once again significantly lower in the ESS group compared to dupilumab (*p* < 0.001) and mepolizumab groups (*p* < 0.001) [10].

It is important to consider the results of the study from Campion et al. contained in this current publication in the context of the above study from Miglani et al. Though the investigators in Campion et al. have shown that NPS improvement is comparable in dupilumab patients with both limited and heavy polyp burdens, we should remember that ESS was ultimately shown by Miglani et al. to offer significantly lower NPS compared to dupilumab.

Complete endoscopic sinus surgery offers some distinct advantages over biologics in terms of long-term polyp control. Perhaps the greatest advantage, in our experience, is the creation of favorable anatomy to decrease inflammation and improve access of topic nasal steroids to the sinus mucosa. This creation of favorable anatomy is often able to dramatically decrease the impact of polyps on the functioning of the paranasal sinuses, thus decreasing symptoms for the patient. Likewise, we must consider patient adherence to treatment. It has unfortunately been our experience that long-term compliance with topical steroid irrigations in patients with CRSwNP is poor. In patients who have undergone *complete* ESS, their optimized anatomy decreases the impact of recurrent polyps and allows for in-office intervention in symptomatic cases of recurrence (ie polypectomy, placement of drug-eluting stents). Non-compliant patients with biologics could expect to see a rapid recurrence of symptoms, given their unaltered anatomy.

### Cost

With current evidence demonstrating that ESS offers the same or *better* performance as biologics, including dupilumab, we should now consider the relative costs of these two treatment modalities.

As of the date of this publication, the cost per course of dupilumab is $31,154 for the first year of therapy, followed by $30,000 per year for each subsequent year [11]. It is too early to be certain, but all current evidence indicates that dupilumab and other biologics would need to be continued indefinitely for the beneficial effects to continue. Indeed, the major clinical trials investigating the efficacy of omalizumab on CRSwNP found that, when treatment was stopped at 52 weeks, patients experienced a gradual worsening of symptoms [12]. The financial implications of such an expensive and long-term therapy are staggering. According to the Centers for Disease Control (CDC), life expectancy for an American man in 2023 is 76.4 years. If we consider a 26 year-old male with CRSwNP, dupilumab administered over the ensuing 50 years would currently be estimated to cost $1.5 million, assuming no change in cost over time (a significant assumption).

In 2021, Scangas et al. performed a cost utility analysis of dupilumab versus endoscopic sinus surgery for CRSwNP [13]. They utilized Quality Adjusted Life Years (QALY) which equate to one year of perfect health. For example, if a drug improves a patient’s quality of life to the point of perfect health for 6 months of a year, this equals 0.5 QALY. Likewise, if a drug improves their quality of life by 50% for one year, this also equates to 0.5 QALY. Generally speaking, in the United States, health economists accept $150,000 to $200,000 per QALY as the cutoff for a reasonable treatment. This cost per QALY is also referred to as the incremental cost effectiveness ratio (ICER).

In their well-designed cohort study, Scangas et al. [13] matched 197 patients who underwent ESS to 293 patients from the prior Sinus 24 and Sinus 52 studies of dupilumab. SNOT-22 scores were compared in both cohorts. The investigators utilized a decision-tree analysis and a 10-state Markov model to assess event probabilities over a 36-year time horizon [13]. The primary outcome was ICER [13].

The results were remarkable, if not completely surprising. The ESS strategy yielded a cost of $50,436.99 and produced 9.80 QALYs (ICER = $5145.63 per QALY) [13]. The dupilumab strategy, on the other hand, yielded a much higher expected cost of $536,420.22 and offered a lower 8.95 QALYs (ICER = $59,935.22 per QALY) [13]. Though the dupilumab ICER still falls within the acceptable limits outlined by health economists, there is clearly a tremendous difference in cost between ESS and dupilumab with a *superior* yield of QALYs with ESS. Indeed, when examining the results of this study, the investigators determined that the annual cost of dupilumab would have to decrease to $855 to match the ICER of ESS [13]. With a current annual price of $30,000, the cost of dupilumab would therefore have to decrease by 97.15% to become an economical alternative to ESS.

Naturally, patients are not bearing the direct burden of the cost of dupilumab. Insurance coverage of dupilumab, in our experience, has been steadily improving. Such costly medications, however, drive up healthcare costs indirectly for all patients in the US. In universal healthcare systems, the economic burden of biologics may be substantial enough to dramatically limit their availability. In developing nations, it is incomprehensible that biologics would be used when a superior and more economical surgical alternative exists.

### Adverse effects

Initial safety and efficacy data for dupilumab in regard to CRSwNP was published in The Lancet in 2019, following completion of a pair of phase 3 clinical trials [3]. This initial data highlighted nasopharyngitis, worsening of nasal polyps/asthma, headache, epistaxis, and injection-site reactions as the most frequent adverse events [3]. Interestingly, reports of conjunctivitis did not feature prominently in the results of these CRSwNP trials, though trials performed for atopic dermatitis noted a higher incidence of mild-to-moderate conjunctivitis [14]. This discrepancy is thought to be due to the higher rate of conjunctivitis in patients with severe atopic dermatitis [14]. Regardless, this does raise questions about other comorbid conditions which could eventually provoke unexpected responses to biologics. Long-term data is essentially limited to omalizumab, and we must be prepared for the possibility of encountering unexpected adverse events related to biologics in the future. Who among us would have expected ranitidine to lead to elevated cancer risk or montelukast to receive a black box warning for suicidal ideation? While this should not necessarily deter us from utilizing biologics, we must consider that we are starting a patient on a relatively new drug with powerful anti-inflammatory effects which they could be using for decades. Accordingly, we feel that it is important to discuss the potential for unforeseen adverse effects with patients.

### Current practice guidelines

Based on the above considerations and the considerable experience and expertise of their authors, Han et al. have released a multidisciplinary consensus treatment algorithm for management of CRSwNP [15]. This study incorporates input from both otolaryngologists and allergists from across the United States. Their algorithm offers biologics in patients whose symptoms recur after *complete* ESS, those with contraindications for surgery, patients with poorly-controlled asthma despite standard therapy / oral steroid-dependent asthma, or patients who decline surgery as part of a shared decision-making process [15]. They specifically advise against the use of biologics in patients with light polyp burden or minimal symptoms [15]. The most recent International Consensus Statement on Allergy and Rhinology: Rhinosinusitis (ICAR: Rhinosinusitis 2021) from The American Rhinologic Society and the American Academy of Otolaryngic Allergy lists dupilumab as a “Recommendation,” stating that it “May be considered for patients with severe CRSwNP who have not improved despite other medical and surgical treatment options [16]”.

The European Forum for Research and Education in Allergy and Airway Diseases (EUFOREA) released their consensus statement on the use of biologics in CRSwNP in 2019, around the same time that the drug was being approved for use in the same patient population in the US [17]. EUFOREA took a slightly different approach to their treatment guidelines, setting forth a list of criteria for biologics. These criteria include evidence of type 2 inflammation, systemic corticosteroids ≥ twice per year, significantly impaired quality of life, significant loss of smell, and diagnosis of comorbid asthma [17]. Patients who undergo surgery need *three* of these criteria to qualify for biologics [17]. Patients without prior surgery need *four* of the above criteria [17]. The authors of those guidelines stipulate that biologics should only be considered in non-surgical patients with severe asthma^17^.

## Conclusions

Biologics, including dupilumab, are very effective medications when used in the correct circumstances. These drugs have led to remarkable improvements in the quality of life of patients with conditions like asthma and atopic dermatitis who do not respond to standard treatments. As excitement grows over the use these drugs and the list of indications expands, however, it is vital to consider the use of biologics for patients with CRSwNP in the context of current regulations, comparable efficacy of surgery, cost, and consensus guidelines. We remain optimistic about the use of biologics, including dupilumab, for recalcitrant CRSwNP, but urge caution when considering biologics as a first-line therapy or when viable revision surgical alternatives exist.

Current regulations offer some guidance to proper use of biologics in CRSwNP. As noted above, however, regulations can be somewhat vague and can change quickly. We acknowledge that regulations are meant to be altered over time, as our understanding of disease processes evolves. That said, we urge careful consideration of all available treatments and strenuous comparison of alternatives (ie. ESS and steroids) before opening the figurative flood gates of first-line use of biologics with CRSwNP. More useful are current treatment guidelines and consensus statements, which add the weight of the vast experience and expertise of their authors to the discussion.

It is also vital for all healthcare providers to understand that available literature shows endoscopic sinus surgery (ESS) to be *at least* as effective as biologics in controlling symptoms of CRSwNP and *more* effective at reducing objective polyp scores. While ESS may be superior at a population level, we must also consider each individual patient. Many patients may be questionable surgical candidates or may have comorbidities like poorly-controlled asthma which could lead them to benefit from a biologic more than surgical intervention.

Perhaps the greatest factor in our recommendation against the use of biologics in patient with mild polyp burden is the cost of treatment. At $30,000 per year, dupilumab is enormously expensive, even when compared to multiple surgeries. When comparing treatment modalities with such similar efficacy, we cannot ignore cost. It is incumbent upon us as physicians to make sure that we are not advocating treatments that our too expensive for our individual patients or that may place undue strain on our overall healthcare system.

Each of the above considerations speaks to the necessity of maintaining an open dialogue with our non-surgical colleagues in allergy/immunology, dermatology and pulmonology, as well as regulators, to ensure that the most appropriate treatment options for CRSwNP do not become lost in the excitement over biologics. Ultimately, there are numerous factors that must be considered for each individual patient, including their surgical candidacy, comorbidities, ability to afford a particular treatment and their personal preferences when making a treatment decision.

We are fortunate to now have ESS, novel topical steroid delivery systems and biologics in our armamentarium for the treatment of CRSwNP. As we proceed into the future of management of CRSwNP, we should do so with both excitement and a healthy degree of caution to ensure that we are providing our patients with the best possible care.

## Data Availability

Not applicable.

## Abbreviations

AERD: Asthma-exacerbated respiratory disease
AFS: Allergic fungal sinusitis
CF: Cystic fibrosis
CRS: Chronic rhinosinusitis
CRSwNP: Chronic rhinosinusitis with nasal polyposis
EC: European Comission
ESS: Endoscopic sinus surgery
EUFOREA: European Forum for Research and Education in Allergy and Airway Diseases
EU: European Union
FDA: Food and Drug Administration
IL: Interleukin
SNOT 22: Sino nasal outcome test (22-item)
Th2: T-helper cell type 2 inflammation
